# Truncation of C-Terminal Intrinsically Disordered Region of Mycobacterial Rv1915 Facilitates Production of “Difficult-to-Purify” Recombinant Drug Target

**DOI:** 10.3389/fbioe.2020.00522

**Published:** 2020-05-29

**Authors:** Monika Antil, Sébastien G. Gouin, Vibha Gupta

**Affiliations:** ^1^Department of Biotechnology, Jaypee Institute of Information Technology, Noida, India; ^2^CEISAM, Chimie Et Interdisciplinarité, Synthèse, Analyse, Modélisation, UMR CNRS 6230, UFR des Sciences et des Techniques, Université de Nantes, Nantes, France

**Keywords:** *Mycobacterium tuberculosis* H37Rv, Rv1915, isocitrate lyase 2, inclusion bodies, solubilization and IDPRs

## Abstract

Availability of purified drug target is a prerequisite for its structural and functional characterization. However, aggregation of recombinant protein as inclusion bodies (IBs) is a common problem during the large scale production of overexpressed protein in heterologous host. Such proteins can be recovered from IB pool using some mild solubilizing agents such as low concentration of denaturants or detergents, alcohols and osmolytes. This study reports optimization of solubilization buffer for recovery of soluble and biologically active recombinant mycobacterial Rv1915/ICL2a from IBs. Even though the target protein could be solubilized successfully with mild agents (sarcosine and βME) without using denaturants, it failed to bind on Ni-NTA resin. The usual factors such as loss of His6-tag due to proteolysis, masking of the tag due to its location or protein aggregation were investigated, but the actual explanation, provided through bioinformatics analysis, turned out to be presence of intrinsically disordered protein regions (IDPRs) at the C-terminus. These regions due to their inability to fold into ordered structure may lead to non-specific protein aggregation and hence reduced binding to Ni-NTA affinity matrix. With this rationale, 90 residues from the C-terminal of Rv1915/ICL2 were truncated, the variant successfully purified and characterized for ICL and MICL activities, supporting the disordered nature of Rv1915/ICL2a C-terminal. When a region that has definite structure associated in some mycobaterial strains such as CDC 1551 and disorder in others for instance *Mycobacterium tuberculosis* H37Rv, it stands to reason that larger interface in the later may have implication in binding to other cellular partner.

## Introduction

Soluble expression of potential drug targets in heterologous host is the limiting factor for their production in amounts required for their structure function characterization, screening of potential inhibitors and for unraveling the mechanism of inhibition. Although *Escherichia coli* is the most popular choice of host for production of recombinant proteins, however, low or no protein expression, incorrect folding or inclusion body formation (IBs), protein inactivity are some common problems during expression in this workhorse organism. Some of the factors responsible for these difficulties are high rates of transcription and translation processes, codon bias, absence of posttranscriptional modification in *E. coli*, unfavorable environment of bacterial cytoplasm for the formation of disulphide-bonds resulting in misfolding/unfolding of the proteins and ultimately leading to protein instability, aggregation and accumulation of the recombinant protein as IBs (Vincentelli et al., 2003; Zhang et al., 2004; Choi et al., 2006; Rosano and Ceccarelli, 2014).

IBs are the pool of partially folded or misfolded proteins which are biologically inactive. IBs were well-characterized in terms of their secondary structure and morphology, indicating that they possess a native-like secondary structure which may have biological activity (Bowden et al., 1991; Oberg et al., 1994; Przybycien et al., 1994). IBs accounts for the 25% of total cellular protein and are enriched in the recombinant protein as opposed to other protein of *E. coli*. In fact, if functionally active protein can be recovered from IBs, then their formation is advantageous as it provides a method for isolation of highly purified protein by (i) isolating IBs from the bacterial cytoplasm, (ii) solubilizing them by using denaturing agents such as urea and guanidine hydrochloride, followed by (iii) refolding via removal of denaturing agents to recover bioactive protein (Rudolph and Lilie, 1996; Vallejo and Rinas, 2004). Of these, solubility and refolding are the two critical steps which affect the time and cost of protein recovery, and thus determine the overall yield of active protein (Rudolph and Lilie, 1996; Burgess, 2009). Generally, use of high concentration of denaturing agents such as urea and guanidine hydrochloride in presence of reducing agent is the most commonly process for solubilization of IBs. However, the high concentration of detergents disrupts the complete secondary structures of the protein which may lead to the aggregation of the protein during refolding process. This problem is overcome by using mild solubilizing agents such as lower concentration of detergents, alcohols, DMSO, high pH, reducing agents (Khan et al., 1998; Process for solubilization of recombinant proteins expressed as inclusion body, 2003; Mohan Singh and Kumar Panda, 2005).

**Figure 1:**
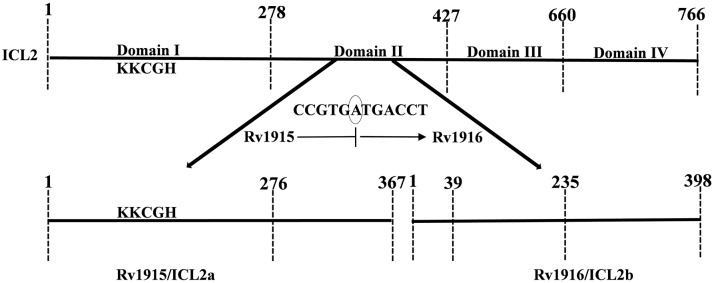
Mapping of full length (*Mtb* strain CDC 1551) and split (*Mtb* strain H37Rv) ICL2. The unique eukaryotic domain II (278–427) in ICL2 of Erdman and CDC1551 strains is divided into two overlapping ORFs namely, Rv1915 (278–367) and Rv1916 (1–59 residues) in H37Rv strain (shown by bold arrows), a consequence of translational coupling phenomenon. Presence of circled nucleotide “A” in the reading frame, results into translation of TGA into stop codon and termination of upstream *rv1915*. The ribosome that has just finished is in a position to initiate translation with ATG start codon for the downstream *rv1916*. The ICL signature motif (KKCGH) present in domain I of both are also shown in the figure.

Mycobacterial infections, are the major concern for public health due to the emergence of drug resistant strains of the pathogen. During its persistence phase *Mtb* resides inside the granulomas which are rich in even and odd chain fatty acids. Activation of glyoxylate pathway allows the pathogen to utilize acetate or propionate (degradation product of fatty acids) as carbon sources for its growth (Bloom, 1994; McKinney et al., 2000). The two important enzymes of this pathway are Isocitrate lyase (ICL) and Malate synthase (MS), the former encoded by 2 genes (smaller *icl1* and larger *icl2*/*aceA*) and the later by *aceB*, respectively. Here it may be helpful to point out for readers that the term “*ace*” came up because the genes that encode for MS, ICL and isocitrate dehydrogenase kinase/phosphatase form an operon aceBAK in *E. coli* (Chung et al., 1988) that functions in acetate utilization. However, operonic arrangement of these genes is not true in all organisms and therefore annotating such genes as “*ace*” is a misnomer and confusing. Specially, in case of *Mtb* H37Rv, the two *icls* that together play an important role in pathogenesis and persistence of the bacterium, are annotated as *icl1* and *aceA* in literature (Cole et al., 1998; Höner Zu Bentrup et al., 1999; Muñoz-Elías and McKinney, 2005). Due to presence of a stop codon in between, the larger *aceA* (766 residues in *Mtb* strain CDC 1551) is split into *aceAa/rv1915* (367 residues) and *aceAb/rv1916* (398 residues) in *Mtb* H37Rv strain (Figure 1). The authors suggest that these split genes be termed as *icl2a* (*rv1915*) and *icl2b* (*rv1916*) for clarity and consistency and the same has been followed in the current study. In case of H37Rv, as evident from sequence mapping of full length and spilt versions of ICL2, ~90 residues involved in the formation of domain II are present in Rv1915/ICL2a, whereas the rest of the 59 residues of domain II are present in Rv1916/ICL2b (Figure 1). Literature documents *Mtb* ICLs to be novel antitubercular drug targets (Wang et al., 2011). The crystal structure of ICL1 (Rv0467), determined in 2,000 by Sharma et al. (2000), was a momentous discovery for structure-based drug designing against *Mtb*. However, although some inhibitors have been reported against *Mtb* Rv0467/ICL1, but no drug is available till date to treat persistent *Mtb*. The possible reasons for this failure are the undiscovered roles of split ICL2 (Rv1915 and Rv1916) that may assist the pathogen to survive in granulomas. In a recent study we reported structure function insights into Rv1916/ICL2b (Antil et al., 2019), but Rv1915 is yet to be characterized. This study reports the difficulties encountered in obtaining soluble expression of the target protein in heterologous host *E. coli* BL21 (DE3) and mainly focuses on strategies adopted for recovery of active Rv1915/ICL2a.

## Materials and Methods

### Chemicals

All chemicals used in this study are commercially available except 2-Methylisocitrate which was synthesized in the laboratory of Dr. Sébastien Gouin, University of Nantes France. The genomic DNA of *Mycobacterium tuberculosis* H37Rv, Luria-Bertani (LB) medium for bacterial growth and Isopropyl β-D-1-thiogalactopyranoside (IPTG) were purchased from Hi-Media Laboratories, India. Primers used for gene amplification were synthesized through Eurofins Genomics India Pvt. Ltd. Restriction enzymes (NheI and HindIII), Alkaline Phosphtase and T4 DNA Ligase were procured from Fermentas, US. DL-Isocitric acid and Phenylhydrazine were obtained from Sigma (India).

### Cloning of *rv1915*

The gene coding for *Mtb* H37Rv Rv1915/ICL2a was amplified by Polymerase chain reaction using a pair of gene specific primers listed in Table 1 from genomic DNA of *Mtb* H37Rv DNA. For directional cloning of the insert DNA, recognition sites for NheI and HindIII restriction enzymes were designed into the 5' end of the forward and reverse primers, respectively (underlined in the Table 1). In addition, to facilitate the target purification, His_6_-tag was incorporated either in the forward primer for N-terminus tag or in the reverse primer for C-terminus tag (highlighted in bold letters in the Table 1). The PCR reaction mix comprised of 1x Taq buffer, 10 ng/μl of genomic DNA, 20 pmoles of each forward and reverse primers, 200 μM dNTPs mix and 4:1 ratio of Taq (Geno biosciences):Pfu polymerase (Fermentas). The standardized PCR cycle for all the three constructs was: initial denaturation at 95°C for 5 min, denaturation at 95°C for 1 min, annealing at 65°C for 1 min and extension at 72°C for 1 min. These conditions were repeated for 30 cycles before a final extension at 72°C for 10 min. The amplified insert and pET-21c expression vector (Novagen) were digested with NheI and HindIII restriction enzymes at 37°C for 1–2 h. The vector and insert were then ligated using T4 DNA ligase followed by the transformation of *E. coli* DH5α with the ligated product. The cells were plated on LB agar plate containing 100 μg/ml ampicillin. Random colonies were picked from the plate and inoculated in 3 ml LB broth supplemented with ampicillin (100 μg/ml). Plasmid was isolated from each of the colonies by alkaline lysis method (Sambrook and Russell, 2006) followed by double digestion with NheI and HindIII restriction enzymes. All the digested products were analyzed by running on 1% agarose gel.

**Table 1:** List of primers used for preparing different constructs of Rv1915/ICL2a.

**S. No**.	**Name (details) of the construct**	**Primer**	**Primer sequence (5^**′**^ to 3^**′**^)**
1	His_6_-Rv1915 (FL Rv1915 with His_6_ tag at N-terminal)	FP	5'-gatttagctagc**catcaccatcaccatcac**gccatcgccgaaacggacaccg-3′
		RP	5′-gatttaaagctttcaggcccgcgtcgtcctc-3′
2	Rv1915-His_6_ (FL Rvk with His_6_ tag at C-terminal)	FP	5'-gatttagctagcgccatcgccgaaacggacaccg-3′
		RP	5′-gatttaaagctttcaggcccgcgtcgtcctccgcgccgagaaggaacggctg-3′
3	Rv1915Δ35CT- His_6_ (Rv1915 with 35 residues deleted from C-terminal)	FP	5'-gatttagctagcgccatcgccgaaacggacaccg-3′
		RP	5′-gatttaaagctttcaagccgaaaatgccttggcgctccgcgccgagaaggaacgg-3′
4	Rv1915Δ90CT- His_6_ (Rv1915 with 90 residues deleted from C-terminal)	FP	5'-gatttagctagccatcaccatcaccatcacgccatcgccgaaacggacaccg- 3′
		RP	5′-gatttaaagctttcagtgatggtgatggtgatgcgcgccgagaaggaacggctg-3′

*FL, Full length; FP, Forward Primer; RP, Reverse Primer*.

### Expression and Cellular Localization of Rv1915/ICL2a

For expression studies the *E. coli* BL21 (DE3) competent cells were transformed with recombinant plasmid. The transformed cells were plated onto LB agar plate containing 100 μg/ml ampicillin and incubated at 37°C overnight. A single colony was inoculated in 5 ml of LB broth supplemented with 100 μg/ml ampicillin and incubated overnight at 37°C with continuous shaking at 200 rpm. 50 μl of this primary culture was then transferred into 5 ml of LB broth containing 100 μg/ml ampicillin and incubated with shaking at 37°C till the OD of culture at 600 nm was 0.5–0.6. The expression of recombinant protein was induced with IPTG concentration, temperature and time as indicated at relevant positions. The culture was harvested by centrifugation at 5,000 rpm for 10 min at 4°C. For analysis of protein induction, cell pellets were directly resuspended in 50 μl of 5X SDS loading dye containing 50 mM Tris-HCl pH 8, 0.25% β-mercaptoethanol (βME), 1% SDS, 10% glycerol and 0.04% Bromophenol blue. On the other hand, for determining localization of the expressed protein, cell pellet of 1 ml culture was lysed by mixing the pellet with 200 μl of 50 mM Sodium Phosphate buffer pH 8, comprising 300 mM NaCl and 20 mg/ml lysozyme, followed by incubation on ice for 15 min and sonication (pulse: 5 s ON and 5 s OFF at 40% amplitude) using ultrasonic water bath (Citizen). After lysis, centrifugation at 12,000 rpm for 15 min segregated the soluble (supernatant) and insoluble fractions (pellet) of the total cell lysate. All samples were boiled at 100°C for 10 min in 1X SDS loading dye before subjecting to 10% SDS-PAGE for expression analysis. For visualization of proteins, gels were stained in 0.25% Coomassie Brilliant Blue R-250 and then destained in 30% (v/v) methanol in water with 10% (v/v) acetic acid solution.

### IBs Isolation and Solubilization of Rv1915/ICL2a

The induced *E. coli* cell culture (1L) was harvested by centrifugation at 5,000 rpm for 10 min at 4°C. The cell pellet was resuspended in 40 ml of 50 mM Tris buffer pH 8.5 containing 5 mM EDTA and 1 mM PMSF. The cells were lysed by sonication on ice for a total time of 20 min (1-min burst and 1-min OFF time) and centrifuged at 15,000 rpm for 30 min at 4°C. The pellet thus obtained was washed with wash buffers A (50 mM Tris pH 8.5, 5 mM EDTA, 1 mM PMSF and 2.5% Triton X-100) and B (50 mM Tris pH 8.5) for three repeated cycles of sonication and centrifugation in each buffer. Finally, the pellet of IBs was dissolved in 2 ml of Milli-Q water and processed for solubilization. In order to obtain bioactive Rv1915, six different solubilization buffers (Table 2) containing mild solubilizing agents were used. For solubilization of IBs, the 2 ml of purified IBs were equally divided (330 μl) in microcentrifuge tube and diluted to a final volume of 1 ml by adding 670 μl of solubilization buffers. The suspensions were vortexed and incubated at room temperature on an end-to-end rotator for an hour. The solubilized samples were separated from insoluble fraction by centrifugation at 15,000 rpm for 30 min at 4°C and the samples were analyzed on 10% SDS-PAGE.

**Table 2:** List of Solubilization Buffers.

**Buffer code**	**Buffer composition**	**Concentration of Rv1915 (mg/ml)**
SB1	50 mM Tris, 5 mM EDTA, 1 mM PMSF, 20 mM βME, 0.25 M Urea, 0.5% Sarcosine pH 8	2.2
SB2	50 mM Tris, 1 mM PMSF, 20 mM βME, 0.5% Sarcosine pH 8	2
SB3	50 mM Tris, 1 mM EDTA, 1 mM PMSF, 10 mM βME, 0.5 M Urea, 0.25% Sarcosine pH 8	1.85
SB4	50 mM Tris, 1 mM EDTA, 1 mM PMSF, 20 mM βME, 0.25 M Urea, 0.5% Sarcosine pH 8	1.99
SB5	50 mM Tris, 5 mM EDTA, 1 mM PMSF, 20 mM βME, 0.5 M Urea, 0.5% Sarcosine pH 8	2.5
SB6	50 mM Tris, 1 mM EDTA, 1 mM PMSF, 5 mM βME, 0.5 M Urea, 0.5% Sarcosine pH 8	1.85

*SB, Solubilization buffer; βME, β-mercaptoethanol*.

### Protein Quantification Using ImageJ Software

ImageJ is a freely available software (https://imagej.nih.gov/ij/download.html), used to determine the protein concentration from SDS-PAGE gels. This software measures the relative density of each protein band from a selected lane of the gel and plot a graph according to their densities. In order to determine the protein concentration, peak area of the band of interest was calculated and compare protein band with the known concentration. To estimate the protein concentration, known amount of BSA (2–10 μg/μl) was run on 10% SDS-PAGE and the relative density of each band was calculated using ImageJ software. BSA standard curve was then prepared using calculated peak area from the software and plotted against the known concentration of BSA. The standard curve thus prepared was used for determining the concentration of solubilized protein from each buffer.

### Ni-NTA Purification of Rv1915/ICL2a

Standard protocol of Ni-NTA affinity chromatography was used for recombinant protein purification. Induced cell pellet of 100 ml was dissolved in 20 ml of lysis buffer containing 50 mM Sodium Phosphate buffer pH 8, 10 mM Imidazole, 300 mM NaCl, 0.5% Sarcosine, 2 mM βME, 1 mM PMSF and 20 mg/ml lysozyme. The buffer optimized from IBs solubilization experiments was further modified according to the standard buffer composition for Ni-NTA affinity chromatography. For lysis, the cell suspension was subjected to sonication for a total time of 10 min which consisted of 10 s ON and 10 s OFF cycles. After sonication, the cell lysate was centrifuged at 12,000 rpm for 30 min to remove the cell debris and the clear lysate was loaded on to 0.5 ml of Ni-NTA column pre-equilibrated with equilibration buffer (50 mM Sodium Phosphate buffer pH 8, 300 mM NaCl and 10 mM Imidazole). After the binding period of an hour, the column was washed with washing buffer A (50 mM Sodium Phosphate buffer pH 8, 300 mM NaCl, 20 mM Imidazole, 2 mM βME and 1 mM PMSF) and B (50 mM Sodium Phosphate buffer pH 8, 300 mM NaCl, 40 mM Imidazole, 2 mM βME and 1 mM PMSF). The bound protein was eluted from the resin with 250 mM Imidazole buffer, quantified by Bradford protein assay, dialyzed against the storage buffer (20 mM Tris pH 8, 100 mM NaCl, 2 mM βME, 1 mM PMSF and 5% glycerol) and aliquots stored at −80°C.

### ICL and MICL Activity Assays

ICL activity of Rv1915/ICL2a was determined by a coupled assay that monitored the formation of glyoxylate-phenylhydrazone complex at 324 nm, generated because the glyoxylate produced in the reaction was further made to react with phenylhydrazine-HCl. In brief, 1 ml of reaction mixture included 50 mM MOPS buffer (pH 7), 1 mM DL-isocitrate trisodium salt, 6 mM MgCl_2_, and 4 mM phenylhydrazine-HCl and 10 μg protein either from solubilized IBs or total cell lysate. Reaction mixture containing solubilization buffers/lysis buffer without enzyme was used as blank. As the protein sample was not pure, total cell lysate of uninduced sample was also assayed for ICL activity as a negative control. For accuracy in comparative analysis, kinetic experiments were carried out under similar experimental conditions as described for Rv1916/ICL2b (Antil et al., 2019). *Mtb* ICLs, also reported to catalyze 2-methylisocitrate and convert it into pyruvate and succinate (Gould et al., 2006). Therefore, 10 and 15 μg of purified enzyme was used for assaying isocitrate and methylisocitrate activity, respectively.

### Bioinformatics Analysis

Sequences of ICL2s from different strains of *Mtb* were aligned using EMBL-EBI tool- Clustal Omega (https://www.ebi.ac.uk/Tools/msa/clustalo/). All the sequences were retrieved from KEGG Database (https://www.genome.jp/kegg/). Expasy tools (https://www.expasy.org/proteomics/protein_structure) were used to predict the secondary structures (alpha, beta random coils and turns) of Rv1915/ICL2a. The disordered regions of Rv1915/ICL2a were further verified by different online servers namely- Prediction of Amyloid Structure Aggregation 2.0 (PASTA 2.0 -http://protein.bio.unipd.it/pasta2/) server, Predictor of Natural Disordered Regions (PONDER - http://www.pondr.com/) and Protein Disorder Prediction Server (PrDOS - http://prdos.hgc.jp/cgi-bin/top.cgi). These servers are freely available and predict the disordered regions of a given protein using its amino acid sequence. PASTA 2.0 predicts the formation of amyloids due to self-aggregation of the given protein using its energy function (Walsh et al., 2014). PONDER uses composition, complexity and hydropathy index of amino acid sequence of a protein to find the disordered regions (Peng et al., 2005). Similarly, PrDOS calculate probability of every amino acid in a protein of being unstructured/disordered (Ishida and Kinoshita, 2007). The quaternary model structure of Rv1915/ICL2a was generated by GalaxyWeb online server (http://galaxy.seoklab.org/cgi-bin/submit.cgi?type=HOMOMER) (Ko et al., 2012)

## Results

### Expression and Localization of Rv1915/ICL2a as IBs

**Figure 2:**
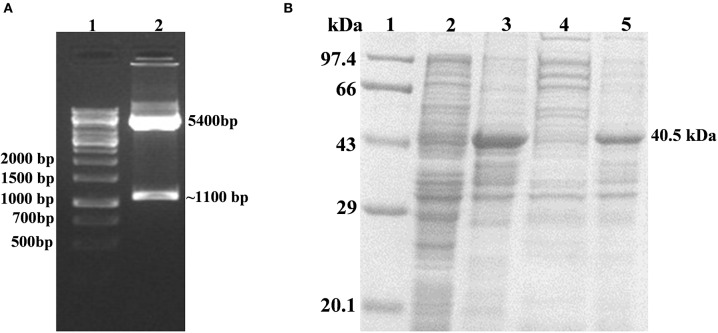
Cloning, Expression and Localization of Rv1915/ICL2a with N-terminal His_6_ tag: **(A)** Confirmation of cloning of Rv1915/ICL2a in pET-21c by double digestion with restriction enzymes NheI and HindIII: Lane 1- 1kb DNA ladder; Lane 2- positive clone of Rv1915 **(B)** Expression and localization of Rv1915: Lane 1- Medium range protein marker; Lane 2- Total cell lysate of uninduced sample; Lane 3- Total cell lysate of induced sample; Lane 4- Soluble fraction; Lane 5- Insoluble fraction.

The successful cloning of *His*_6_*-rv1915* in pET-21c was confirmed by double digestion with restriction enzymes NheI and HindIII. The fall out of 1.1 kb confirms the presence of insert *rv1915/icl2a* in pET-21c vector (Figure 2A). The expression of recombinant His_6_-Rv1915, induced with 1 mM IPTG for 16–18 h at 18°C was analyzed on 10% SDS-PAGE. Figure 2B, confirms the expression of Rv1915/ICL2a at their expected size i.e., ~ 40.5 kDa. Unfortunately, accumulation of induced protein in the insoluble pellet/IBs (Figure 2B, lane 4) leave negligible or no Rv1915 protein in the soluble fraction of the lysate (Figure 2B, lane 5). Despite extensive efforts involving variation in the induction temperature and IPTG concentration, media optimization, addition of osmolytes/chaotropes/additives in the culture media during cell growth etc., soluble expression of the induced protein could not be achieved (Figures S1–S3).

### Recovery of Bioactive Rv1915/ICL2a From IBs

**Figure 3:**
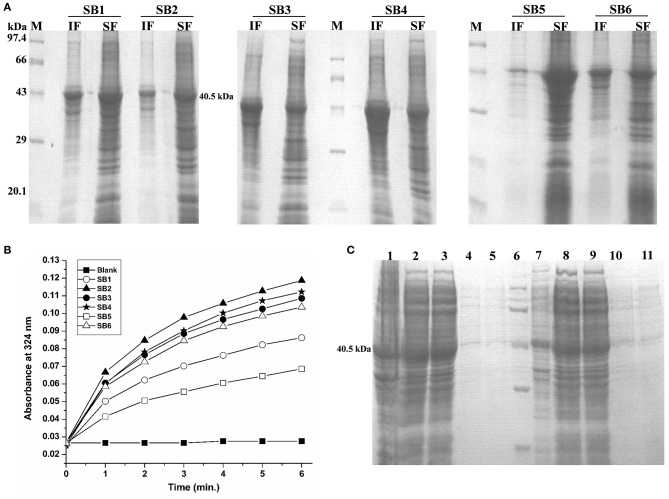
Standardization of solubilization buffer and purification of Rv1915/ICL2a **(A)** SDS-PAGE analysis for solubilization of His_6_-Rv1915 IBs in different SBs: Lane M: Medium range protein marker; Lane IF: Insoluble fraction; Lane SF: Soluble fraction. Composition of all SBs are listed in Table 2; **(B)** Activity assay of Rv1915/ICL2a solubilized from IBs: Increased absorbance at 324 nm with the addition of Rv1915 (extracted from different SBs) indicate formation of glyoxylate phenylhydrazone complex and hence ICL activity; **(C)** Purification of full length His_6_-Rv1915 at N-terminal using SB2 buffer (Lane 1- Insoluble fraction; Lane 2- Soluble fraction; Lane 3- Flow through; Lane 4 and 5- Eluted fractions; Lane 6- Medium range protein marker) and Rv1915-His_6_ at C-terminal (Lane 7- Insoluble fraction; Lane 8- Soluble fraction; Lane 9- Flow through; Lane 10 and 11- Eluted fractions).

IBs of Rv1915/ICL2a were isolated as described in section IBs Isolation and Solubilization of Rv1915/ICL2a and six different solubilization buffers (SBs) varying in concentrations of urea, EDTA, βME and sarcosine were designed for solubilizing inclusion body protein Rv1915. Table 2 represents the composition of SBs and concentration of solubilized His_6_-Rv1915 in respective buffers. SDS-PAGE analysis of IBs solubilization using different buffers is depicted in Figure 3A. Almost all the buffers were able to solubilize the IBs of protein of interest to some extent, with highest concentration (2.5 mg/ml) of His_6_-Rv1915 achieved in buffer SB5, composed of 50 mM Tris pH 8, 5 mM EDTA, 1 mM PMSF, 20 mM βME, 0.5 M Urea and 0.5% Sarcosine (Table 2). In order to select the appropriate buffer for the recovery of bioactive His_6_-Rv1915, activity assay was performed with soluble fraction of His_6_-Rv1915 from each buffer. As lowest activity of His_6_-Rv1915 was observed in SB5 buffer, it was deemed unsuitable (Figure 3B). The highest activity was achieved in buffer SB2 where the solubilizing additive was only 0.5% sarcosine without urea or EDTA. EDTA appears to be more detrimental for the activity of Rv1915 than urea, as reducing the concentration of ETDA to 1 mM in SB4 (but keeping urea same) increases ICL activity almost comparable to the SB2. Furthermore, SB3 and SB6 shows substantial reduction in amount and activity of soluble protein due to the decrease in concentration of sarcosine and βME, respectively (Figure 3A). From all these observation it was concluded that sarcosine and βME plays an important role in solubility and activity of His_6_-Rv1915, so these standardized conditions were employed in purification of His_6_-Rv1915. Unfortunately, even after the solubilization of His_6_-Rv1915 with 0.5% sarcosine, Ni-NTA affinity purification of the protein could not be achieved due to inefficient binding of the protein to the Ni-NTA beads (Figure 3C, lanes 2 & 3). The possible reasons could be inaccessibility/masking of the His_6_-tag due to formation of soluble aggregates of the protein or loss of His-tag due to proteolysis. Alternatively, if proteins possess signal peptide and transmembrane domain at their N-terminus which when liberated will result in loss of tag and therefore reduced binding to Ni^2+^ matrix. The presence of these signal peptides at N-terminus was checked with the online servers - SignalP.4 (http://www.cbs.dtu.dk/services/SignalP-4.0/) (Petersen et al., 2011) and TMHMM (http://www.cbs.dtu.dk/services/TMHMM/) (Sonnhammer et al., 1998; Krogh et al., 2001) and negated the possibility. To overcome the problem of the His_6_-tag being masked or degraded at the N-terminus, recombinant Rv1915-His_6_ was prepared where the His tag was placed at C-terminal of the protein (Figure S4), but the problem still persisted (Figure 3C, lanes 8 & 9). Finally, bioinformatics analysis was performed that provided some clue for resolving the problem.

### Sequence and Structure Analysis of Rv1915/ICL2a

**Figure 4:**
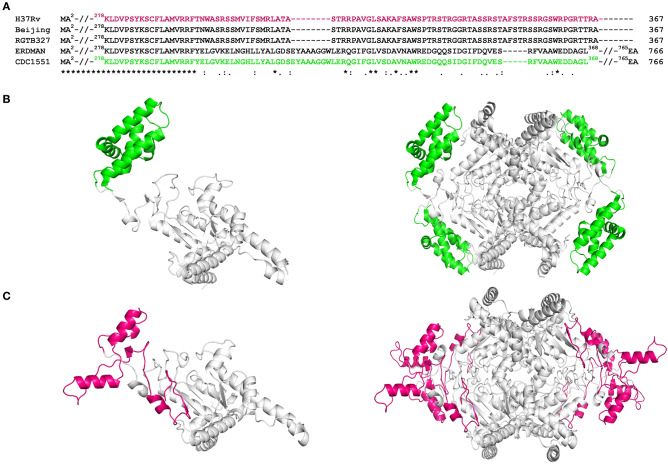
Sequence and structure analysis of Rv1915/ICL2a: **(A)** Multiple sequence alignment of Rv1915/ICL2a and ICL2 from different strains of *Mtb* represents the variation at the C-terminal (residues 278–367) of spilt versions of ICL2s (H37Rv, RGTB327 and Beijing) vs. larger ICL2s (Erdman and CDC1551) (residues 278-368); **(B)** Crystal structure of larger ICL2 from *Mtb* CDC1551 (extracted for residues 1–368 from PDB code 6EDW) illustrates monomer (left) and tetramer (right) highlighting the variable residues (278–368) that form part of domain II in green color; **(C)** Model structure of monomeric (left) and tetrameric (right) Rv1915/ICL2a generated by GalaxyWeb online server shows the disordered nature and different orientation of equivalent C-terminal tail in magenta color. Complete sequence identity between the ICL2s in the region before the variable residues is reinforced by undistinguishable gray color cartoon structures in both the homologs.

Multiple sequence alignment of Rv1915/ICL2a with ICL2s from other *Mtb* strains reveal variability mostly in the C-terminal residues (Figure 4A). This difference was somewhat anticipated, as compared to larger ICL2s (~766 amino acids), H37Rv ICL2 is split in two ORFs where Rv1915 forms the first part and Rv1916 the later. Secondary structure prediction based on the primary sequence of Rv1915 estimated ~41.96% disorder (Figure S5), where out of 72 C-terminal residues 40 of them are random coil (highlighted in the black box). In the united version of ICL2, the equivalent region is comprised of helices, therefore, the splitting of this helical region is increasing disorder at the C-terminal of Rv1915. Further analysis with the PASTA 2.0 server corroborated that the 314–367 residues of Rv1915/ICL2a are disordered and have the propensity for self-aggregation and amyloids formation (Table 3). The software also predicts two additional segments (248–251 and 304–307) with tendency toward parallel aggregation. Two other servers PrDOS and PONDER endorsed the presence of disordered regions at the termini of Rv1915/ICL2a (Figure S6). Specifically, ~35 residues from the C-terminus and ~15 residues from the N-terminus of the queried protein was predicted to be unstructured by all the three servers. *In silico* deletion of either of these in PASTA 2.0 server did not reduce the number of amyloids, which could be achieved only after truncation of ~90 residues (278–367) from the C-terminal end of Rv1915 (Table 3). This 90 residue long C-terminal region encompasses the second aggregation segment (residues 304–307), whose removal appears to reduce amyloid formation. As discernible from multiple sequence alignment (Figure 4A), this section has low similarity with the larger ICL2, reflected in the structural differences as well. The comparison of equivalent/similar structural region of *Mtb* ICL2 (Figure 4B) and the model structure of Rv1915/ICL2a also illustrates the disordered nature of C-terminal 90 residues (colored pink in Figure 4C). Therefore, in order to minimize the probability of the non-specific aggregation of the expressed target, two deletion variants of *rv1915/icl2a* were designed with 35 and 90 residues truncated from the C-terminus.

**Table 3:** Prediction of IDPRs of Rv1915/ICL2a using PASTA 2.0 server.

**S. no**.	**Different variant Rv1915/ICL2a**	**Length of Rv1915/ICL2a**	**No. of amyloids**	**Best energy**
1.	Full length Rv1915	367	2	−5.448
2.	Rv1915 with of 15-residues truncated from N-terminus	352	2	−5.448
3.	Rv1915 with of 35-residues truncated from C-terminus	332	2	−5.448
4.	Rv1915 with of 90-residues truncated from C-terminus	277	1	−5.448

### Effect of C-Terminal Truncation on the Solubility and Activity of Rv1915/ICL2a

**Figure 5:**
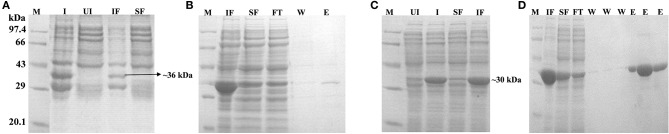
Expression, localization and purification of Rv1915Δ35CT **(A,B)** and Rv1915Δ90CT **(C,D)**: Lane M, Medium range protein marker; Lane I, Total cell lysate of induced sample; Lane UI, Total cell lysate of uninduced sample; Lane IF, Insoluble fraction; Lane SF, Soluble Fraction; Lane FT, Flow through after binding to the Ni-NTA resin; Lane W, Washes with different concentrations of imidazole; and Lane E, Elutions with 250 mM imidazole.

**Figure 6:**
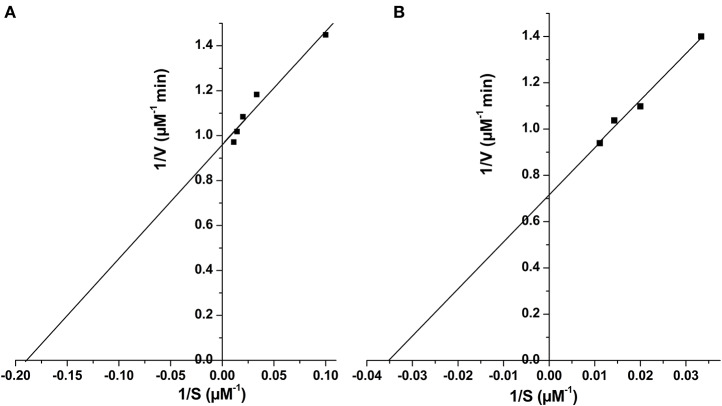
Determination of kinetic parameters of Rv1915Δ90CT using Lineweaver Burk plot for both the substrate, isocitrate **(A)** and methylisocitrate **(B)**.

Two C-terminal truncated variants of *rv1915*, namely, Rv1915Δ35CT-His_6_ and Rv1915Δ90CT-His_6_, were cloned in pET-21c vector using methodology detailed in section Cloning of *rv1915*. Recombinant clones were confirmed by double digestion with the same restriction enzymes (Figure S7). The deletion variants were expressed in *E. coli* BL21 (DE3), induced with 1 mM IPTG (Figures 5A,C) and purified with Ni-NTA affinity chromatography (Figures 5B,D). Out of the two variants, purification could be achieved only for Rv1915Δ90CT-His_6_ due to inept binding of Rv1915Δ35CT-His_6_ to Ni-NTA resin (Figures 5A,B), similar to the problem encountered in the case of full length Rv1915-His_6_ (Figure 3C). Figure 5D shows the profile of Ni-NTA eluted fractions of Rv1915Δ90CT on 10% SDS-PAGE. The total yield corresponded to ~20 mg/l. In order to compare the effect of C-terminal truncation on the functionality of Rv1915/ICL2a, and since purified full length Rv1915 could not be achieved, ICL activity was carried out with crude lysates (Figure S8). The observations show that the C-terminal truncation improves the activity of Rv1915/ICL2a. Kinetic parameters of Rv1915Δ90CT were determined using Lineweaver-Burk plot for both the substrates; isocitrate and 2-methylisocitrate (Figure 6). The kinetic parameters reveal that Rv1915Δ90CT has ~50 fold higher affinity for isocitrate (5.2 μM) than 2-methylisocitrate (279 μM). Calculation of catalytic efficiency turns out to be 0.83 μM^−1^ min^−1^ for isocitrate and 0.0137 μM^−1^ min^−1^ for 2-methylisocitrate, reinforcing faster turnover of isocitrate into glyoxylate and succinate (as compared to conversion of 2-methylisocitrate to pyruvate and succinate). Similar trend was observed for Rv1916/ICL2b (Table 4). However, Rv0467/ICL1 unequivocally displays much higher activity for both the substrates in comparison to Rv1915/ICL2a and Rv1916/ICL2b (Antil et al., 2019).

**Table 4:** Kinetic parameters of recombinant ICLs of *Mtb* H37Rv.

**Enzyme**	**Amount of enzyme (μg)**	**Kinetic parameters**			**References**
		**K_m_ (μM)**	**K_cat_ (min^**−1**^)**	**K_cat_/K_m_ (μM^**−1**^ min^**−1**^)**	
**ICL Activity**					
1915Δ90CT	10	5.22	4.33	0.83	This study
Rv1916	10	13	6.87	0.53	Antil et al., 2019
Rv0467	2	3.25	30.9	9.5	
**MICL Activity**					
1915Δ90CT	15	279	3.84	0.0137	This study
Rv1916	25	300	1.2	0.004	Antil et al., 2019
Rv0467	5	240.9	8.05	0.033	

## Discussion

This work was initiated with an aim to produce Rv1915/ICL2a, an important drug target of *Mtb* H37Rv, in ample amounts for structure function studies. Therefore, Rv1915/ICL2a was cloned in pET-21c vector and expressed in *E. coli* BL21 (DE3) strain but the recombinant protein localized in the insoluble fraction of cell lysate. Protein misfolding/unfolding/formation of insoluble IBs is often a problem during the overexpression of recombinant proteins. Employing different strategies such as reducing IPTG concentration, expression temperature, choice of right expression vector and host strain, optimizing composition of the culture media, co-expression with molecular chaperones, purification from inclusion bodies, etc. may help in overcoming the problem of insoluble expression in *E. coli*. Therefore, different induction temperature, inducer concentration, growth media were explored but none of these increased the solubility of the recombinant protein (Figures S1–S3). Attempt to isolate Rv1915/ICL2a IBs followed by solubilization in mild buffers did yield active protein but could not be purified further as it did not bind to Ni-NTA (Figure 3C). The possible reasons could be masking or degradation of the His_6_-tag. In any case all efforts to obtain soluble Rv1915 in amounts enough for further studies reached a dead end.

Multiple sequence alignment of *Mtb* ICL2a with 766 amino acid long ICL2s show high variability in the region of domain II where the later divided into two ORFs (Figure 4A). It appears that gene duplication in domain II may be responsible for structural divergence and evolution of this split version of ICL2. Fortunately the crystal structure of ICL2 from *Mtb* strain CDC 1551/Oshkosh (PDB code 6EDW), has recently become available (Figure 4B) (Bhusal et al., 2019) and helped in building homology model of Rv1915/ICL2a (Figure 4C). Both secondary structure and 3D model predicted disordered C-terminus Rv1915/ICL2a (residues 278–367) as opposed helical region of long ICL2. For reducing non-specific aggregation due to presence of floppy tails, recombinant DNA technology was employed for generating two variants of Rv1915 where 35 (Rv1915Δ35CT) and 90 (Rv1915Δ90CT) residues from the C-terminal were deleted. Only the later could be purified successfully that exhibited dual ICL and MICL activities, observed first time for *Mtb* H37Rv strain. Dual activity has been reported for the complete *Mtb* strain CDC 1551/Oshkosh ICL2 (Bhusal et al., 2019), but no activity data exists for Rv1915/ICL2a till date. Functional characterization of Rv1915/ICL2a follows our previous study on Rv1916/ICL2b (Antil et al., 2019) and although both Rv1915/ICL2a and Rv1916/ICL2b display dual activities, ICL and MICL activities of both the proteins are much lower than that exhibited by Rv0467/ICL1 (Table 4). Nevertheless, all three *Mtb* ICLs show preference for isocitrate over methylisocitrate.

It may be worth pondering on the biological significance of presence of IDPR in otherwise structured Rv1915. The propensity of these IDPRs to bind to multiple biological partners and their role in cellular activities such as gene replication, transcription, regulation and signal transduction is becoming evident (Uversky, 2013). Under physiological conditions, IDPRs do not have stable three dimensional structure, but they may attain a stable conformation after binding to their biological ligands or other cellular proteins (Dunker et al., 2001, 2002; Uversky, 2019). Disordered structure provides larger surface area for binding to its partner, perform various regulatory roles and have the ability to respond quickly to environmental cues. Specifically, IDPRs of bacterial pathogens can alter the host immune responses either by mimicking host cell signaling components or by forming complexes with proteins of the host cells and thereby disturbing its protein-protein interactions (Marín et al., 2013). It stands to reason that *Mtb* ICL2 (Rv1915 and Rv1916), known to be essential for chronic infection, may be playing similar regulatory role facilitated by IDPR. Recently, 3-dimensional structure of *Mtb* ICL2 (CDC1551 strain) along with Small-angle X-ray scattering analyses and Molecular Dynamic simulations have led to molecular level understanding of its allosteric activation at high lipid concentrations and of its function (Bhusal et al., 2019). Future structural studies of Rv1915 and Rv1916 will provide a better picture of their roles in *Mtb*'s virulence.

## Conclusion

This study reports the cloning and accumulation of recombinant Rv1915/ICL2a as IBs. Although soluble protein could be recovered from these aggregates using βME and sarcosine, however, purification could not be achieved. Amino acid sequence and structure analysis predicted IDPRs in Rv1915/ICL2a, which were further confirmed by *in silico* deletion of the disordered regions and correlation with reduced number of amyloids in the query protein. C-terminal 90 residues deleted recombinant Rv1915Δ90CT could be purified to homogeneity, implementing IDPRs to be responsible for aggregation of Rv1915/ICL2a and deterrent in purification. Presence of IDPR suggests regulatory role for Rv1915/ICL2a by interaction with some cellular partners. Availability of this “difficult to purify” has led to its biochemical characterization and opens venue for structure function studies and inhibitor discovery.

## Data Availability Statement

The raw data supporting the conclusions of this article will be made available by the authors, without undue reservation, to any qualified researcher.

## Author Contributions

MA designed, carried out all the experiments, and compiled the data. SG synthesized 2-methylisocitrate substrate for analyzing MICL activity of Rv1915. VG conceived the idea, procured the extramural funds, analyzed the results, and supervised the study. MA and VG wrote the manuscript.

## Conflict of Interest

The authors declare that the research was conducted in the absence of any commercial or financial relationships that could be construed as a potential conflict of interest.
