# Supplementary material for: Truncation of C-Terminal Intrinsically Disordered Region of Mycobacterial Rv1915 Facilitates Production of “Difficult-to-Purify” Recombinant Drug Target
Source: Front Bioeng Biotechnol. 2020 May 29;8:522. doi: 10.3389/fbioe.2020.00522 (PMC7273500; doi:10.3389/fbioe.2020.00522)
Supplement: Supplementary file 1 [file Data_Sheet_1.doc]

***Supplementary Material***

**Supplementary Figures**


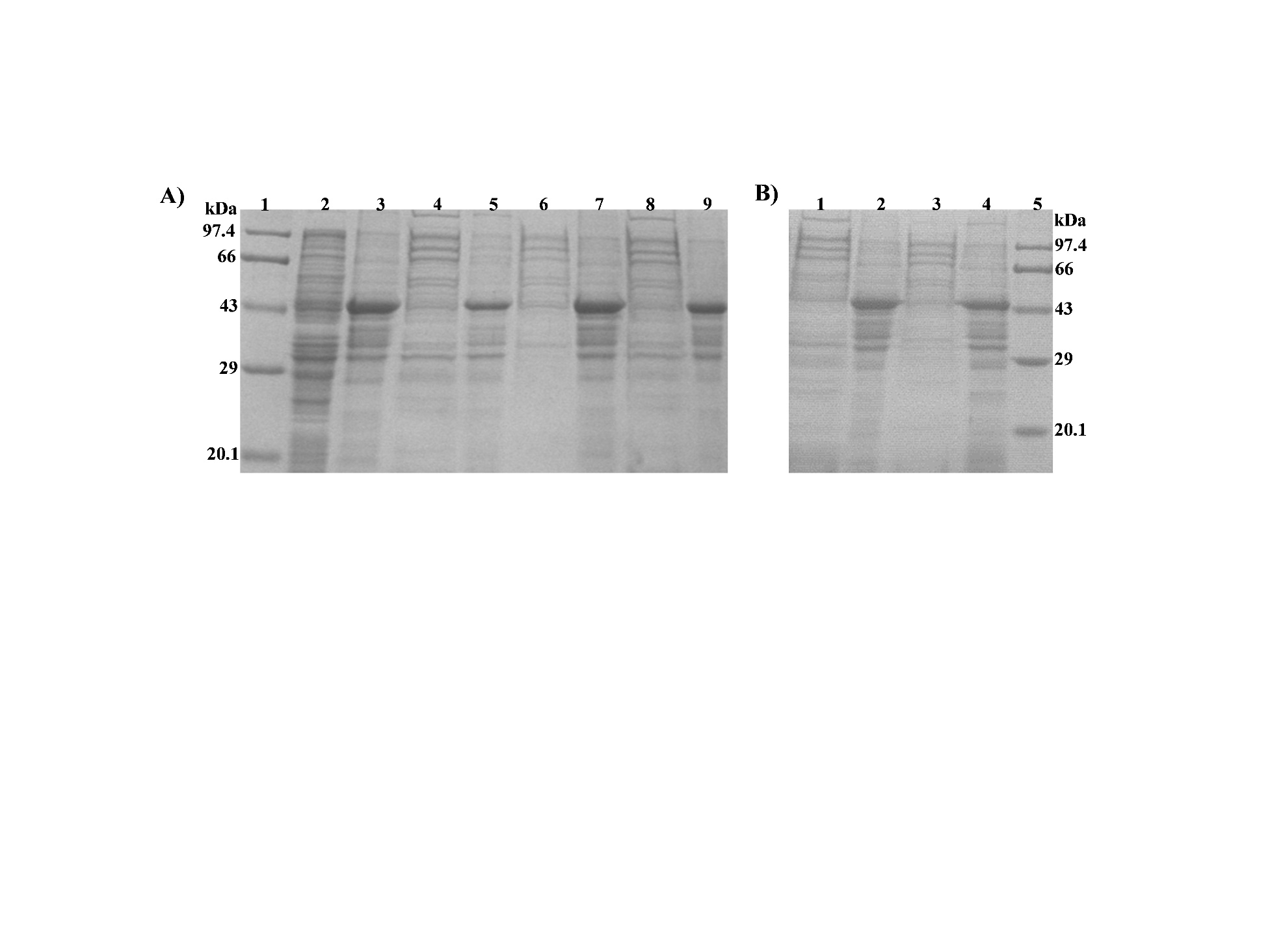
**Optimization of Induction Temperature and IPTG Concentration**

**Supplementary Figure 1:** **(a)** SDS PAGE analysis illustrating localization of His_6_-Rv1915 in the insoluble fractions when induced at different temperature with 1mM IPTG concentration: Lane 1: Medium range protein; Lane 2: Total cell lysate of uninduced culture; Lane 3: Total cell lysate of induced culture; 4, 6 and 8: Soluble fraction of cell culture induced at 18 °C, 37 °C , 25°C respectively with 1mM IPTG; Lane 5, 7 and 9: Insoluble fraction of cell culture induced at 18 °C, 37 °C , 25°C respectively; (b) SDS PAGE demonstrating localization of recombinant Rv1915/ICL2a induced with different IPTG concentration at 18 °C: Lane 1 and 3: Soluble fraction of cell culture induced with 0.5 mM and 0.25mM IPTG respectively; Lane 2 and 4: Insoluble fraction of cell culture induced with 0.5 mM and 0.25mM IPTG respectively.


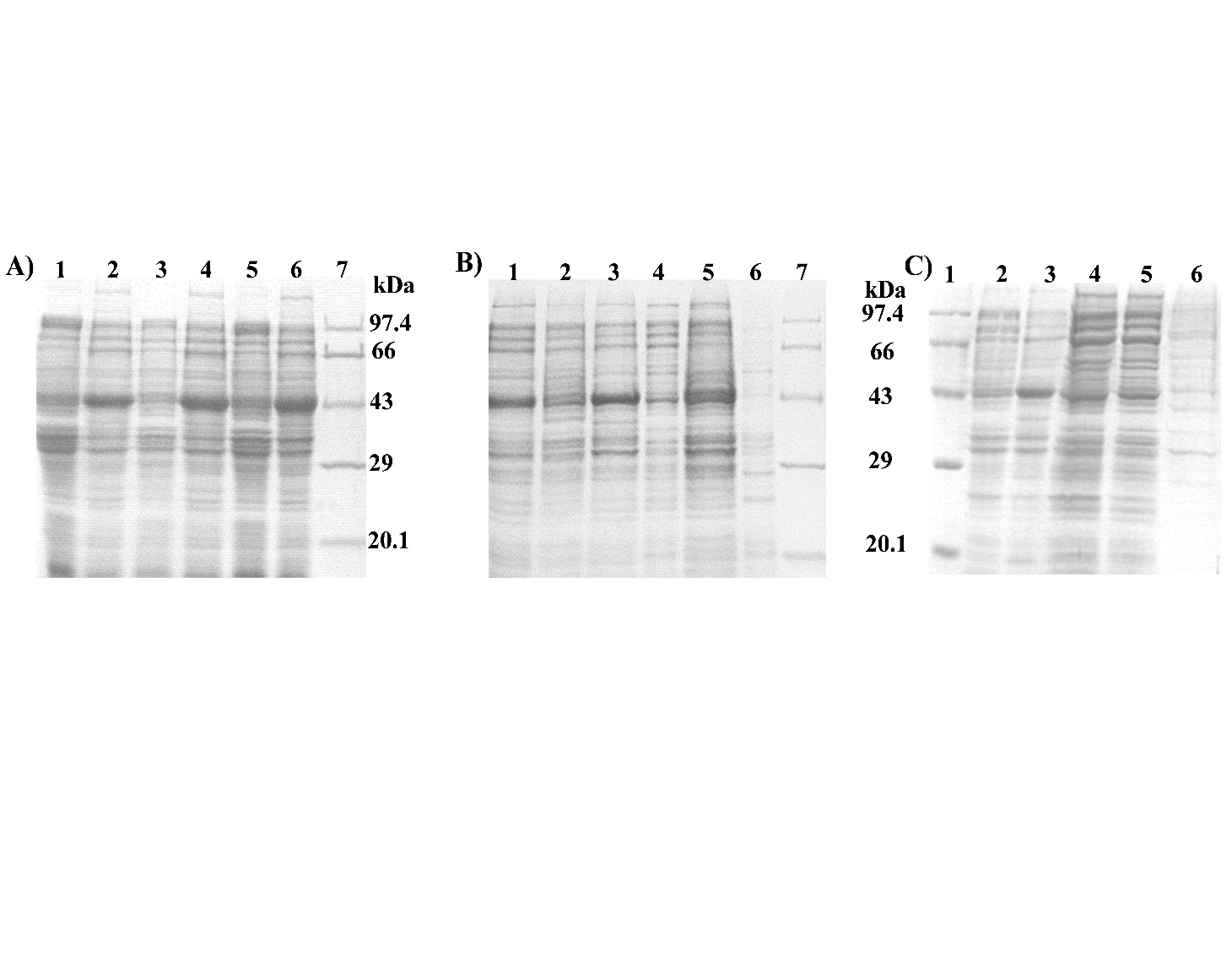
**Media Optimization**

**Supplementary Figure 2:** SDS PAGE analysis of expression and localization of His_6_-Rv1915 in different media at 18˚C induced with 1mM IPTG: (a) Lane 1, 3 and 5: Total cell lysate of uninduced cultures grown in LB broth, Super broth and M9Y respectively; Lane 2, 4 and 6 Total cell lysate of induced cultures grown in LB broth, Super Broth and M9Y respectively. Lane 7: Medium range protein marker (b) Lane 1, 3 and 5: Insoluble fraction of cells grown in LB broth, Super Broth and M9Y respectively; Lane 2, 4 and 6: Soluble fraction of cells grown in LB broth, Super broth and M9Y respectively; Lane 7: Medium range protein marker; a less amount of protein was observed in soluble fraction in case of Super Broth which was further attempted to purify with Ni-NTA affinity chromatography (c) Lane 1: Medium range protein; Lane 2: Total cell lysate of uninduced sample; Lane 3: Insoluble fraction; Lane 4: Soluble fraction; Lane 5: Flow through; Lane 6: Eluted fraction of Rv1915 indicating the lower and non-specific binding on the resin.

**Expression of Rv1915/ICL2a in the presence of Different Osmolytes**

**
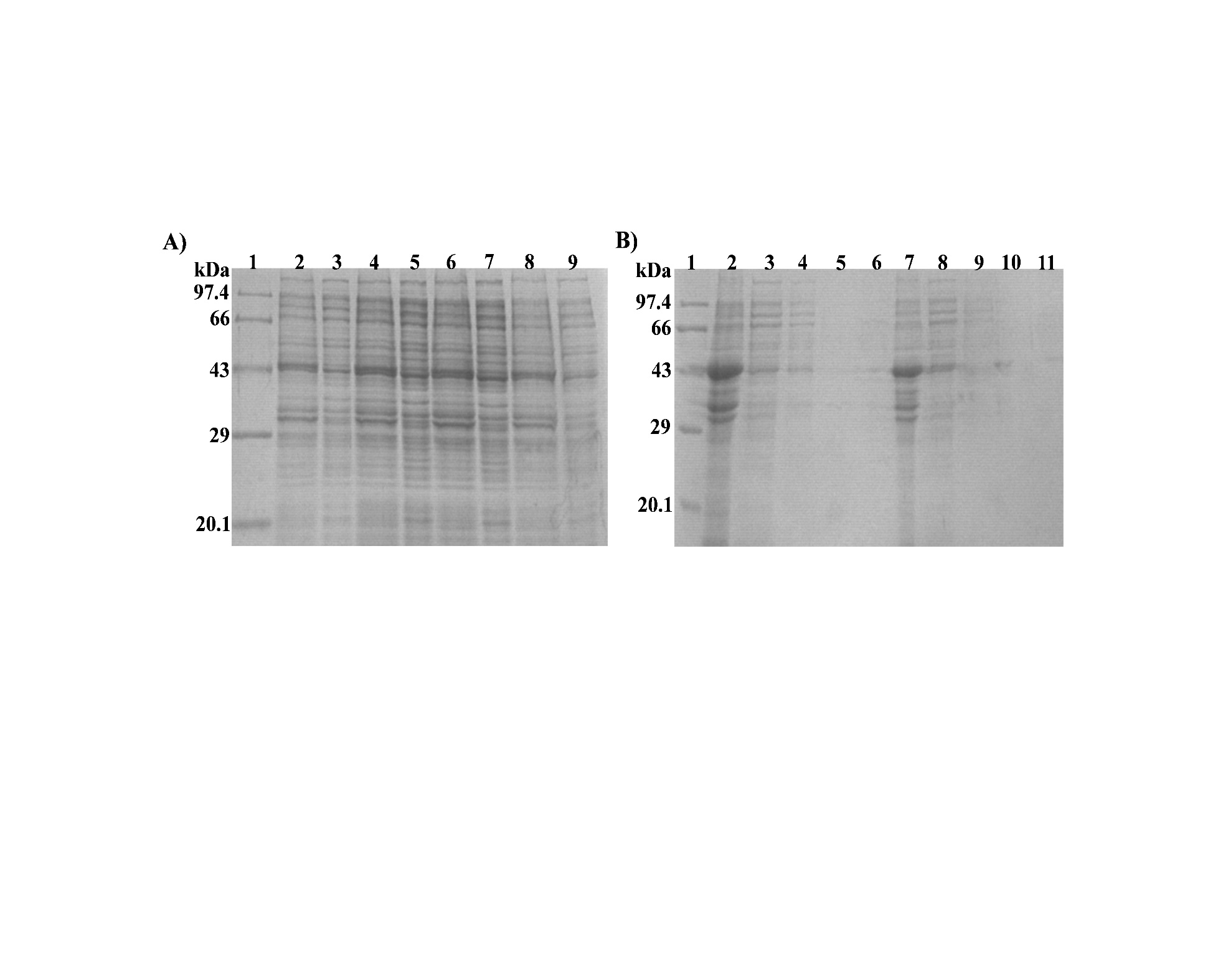
**

**Supplementary Figure 3:** SDS PAGE analysis of expression and localization of His_6_-Rv1915 after addition of various osmolytes during secondary culture induced with 1mM IPTG: (a) Lane 1: Medium range protein; Lane 2, 4, 6 and 8: Insoluble fraction of cells grown in presence of 0.5M NaCl, 0.1% Glycerol, 5mM Glutamic acid and 10% glucose respectively; Lane 3, 5, 7 and 9: Soluble fraction of the cells 0.5M NaCl, 0.1% Glycerol, 5mM Glutamic acid and 10% glucose respectively. Soluble fractions of 0.1% Glycerol and 5mM Glutamic acid were subjected to Ni-NTA affinity purification (b) Small scale purification of Rv1915/ICL2a: Lane 1: Medium range protein; Lane 2: Insoluble fraction; Lane 3: Soluble fraction; Lane 4: Flow through; Lane 5 and 6: Clear lane of eluted fraction of Rv1915 induced in the presence of 0.1% Glycerol showing the no binding of the protein on the resin; Lane 7: Insoluble fraction; Lane 8: Soluble fraction; Lane 9: Flow through; Lane 10 and 11: Eluted fraction of Rv1915 induced in the presence of 5mM Glutamic acid showing the same results as in case of glycerol.

**Cloning expression and localization of Rv1915-His_6_ with His tag at C-terminus**


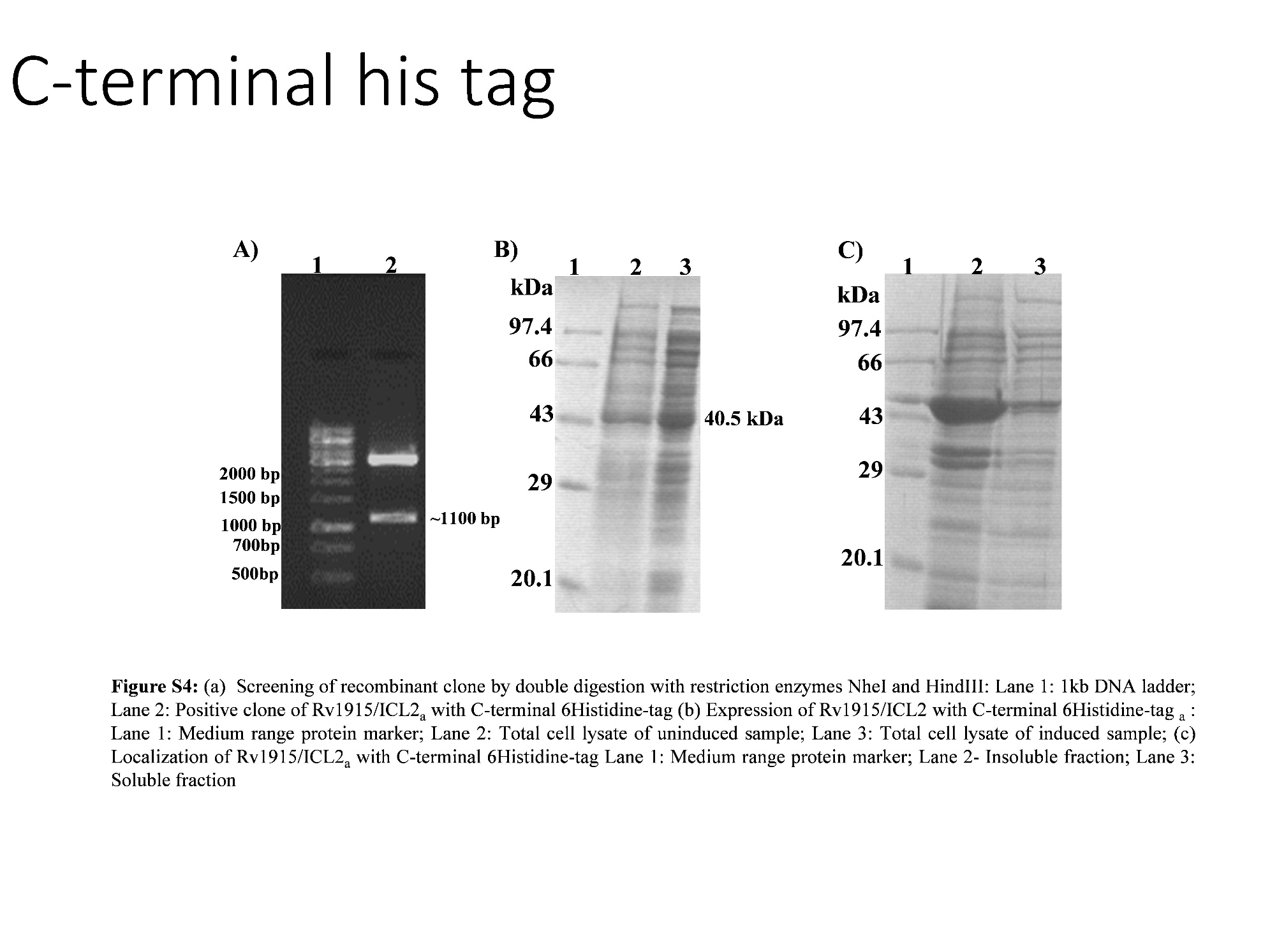


**Supplementary Figure S4:** (a) Screening of recombinant clone by double digestion with restriction enzymes NheI and HindIII: Lane 1: 1kb DNA ladder; Lane 2: Positive clone of Rv1915-His_6_ (b) Expression of Rv1915-His_6_: Lane 1: Medium range protein marker; Lane 2: Total cell lysate of uninduced sample; Lane 3: Total cell lysate of induced sample; (c) Localization of Rv1915-His_6_ showing the accumulation of the protein in insoluble fractions Lane 1: Medium range protein marker; Lane 2- Insoluble fraction; Lane 3: Soluble fraction

**Secondary Structure Prediction of full length Rv1915/ICL2a**

**Supplementary Figure 5:** Secondary Structure prediction of Rv1915/ICL2a by Expasy server. The 72 variable residues of Rv1915, equivalent to the larger ICL2 are represented in the black box

**Disordered Structure Prediction**

**
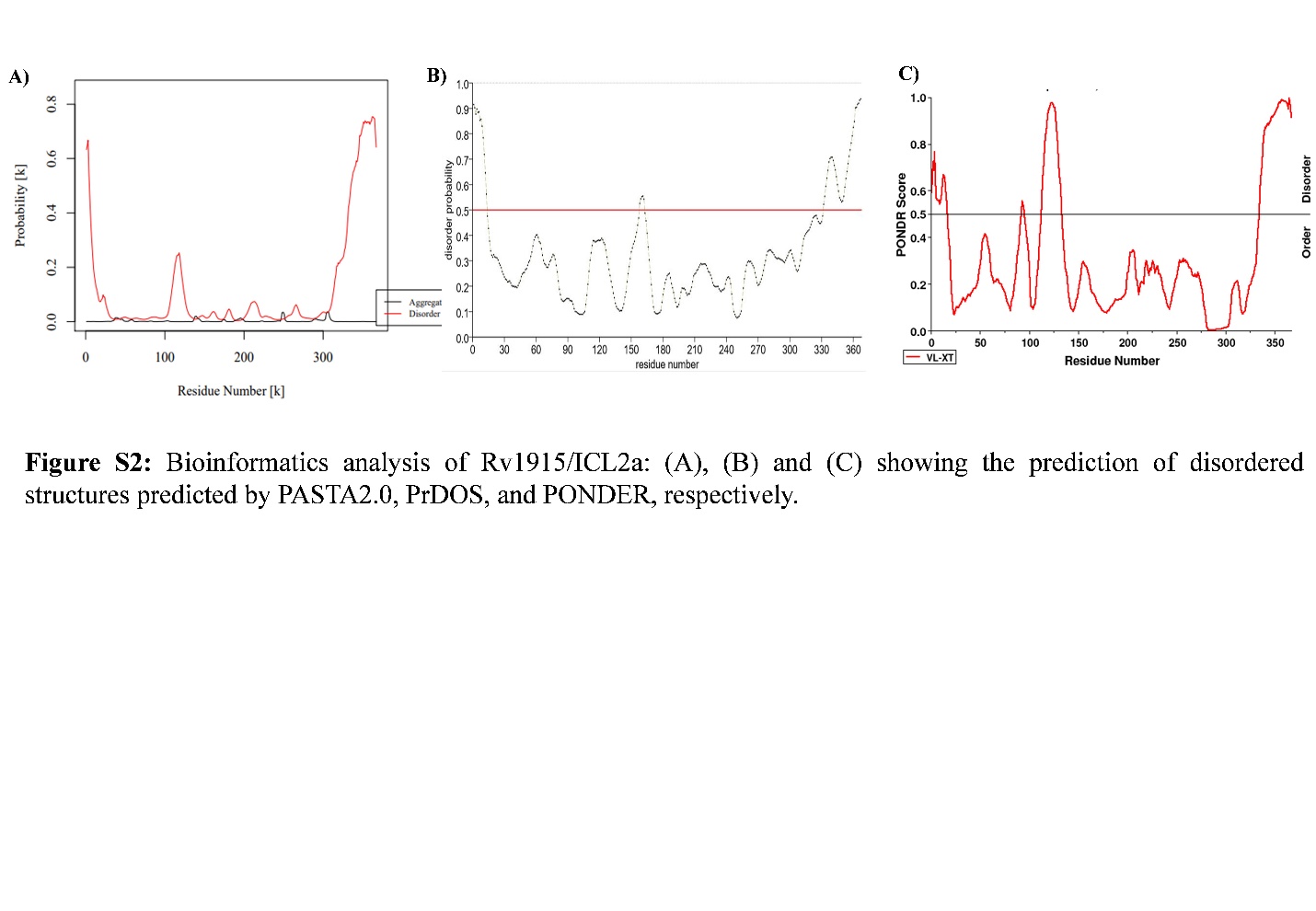
**

**Supplementary Figure 6:** Sequence analysis of Rv1915/ICL2a: (a), (b) and (c) showing the prediction of disordered structures predicted by PASTA2.0, PrDOS, and PONDER, respectively

**Cloning of Truncated Rv1915 with C-terminal His6-tag**

**
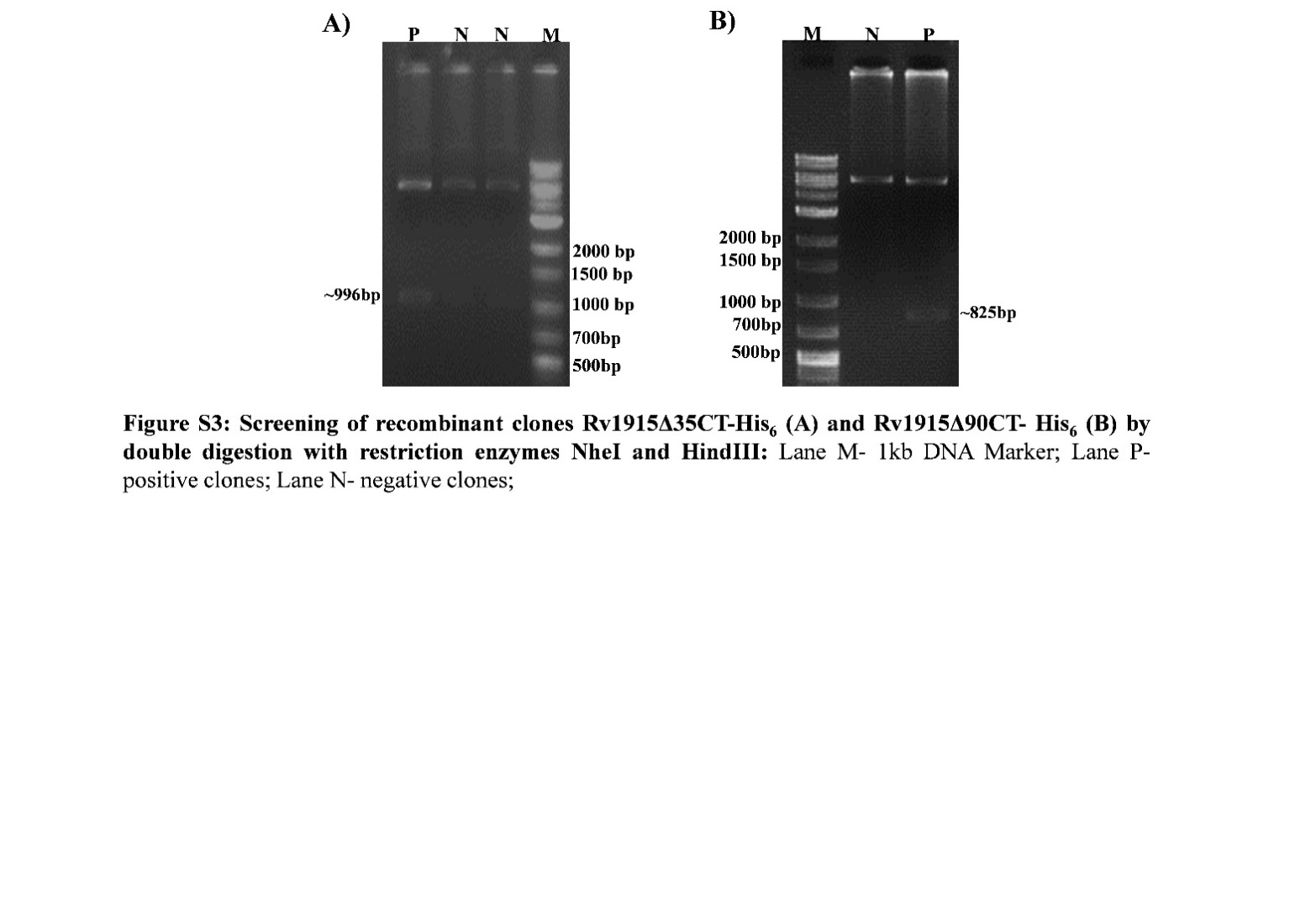
**

**Supplementary Figure 7:** **Screening of recombinant clones Rv1915Δ35CT-His_6_ (a) and Rv1915Δ90CT-His_6_ (b) by double digestion with restriction enzymes NheI and HindIII:** Lane M- 1kb DNA Marker; Lane P- positive clones; Lane N- negative clones

**Comparision of ICL activity of full length Rv1915 and trncated Rv1915Δ90CT variant**

**
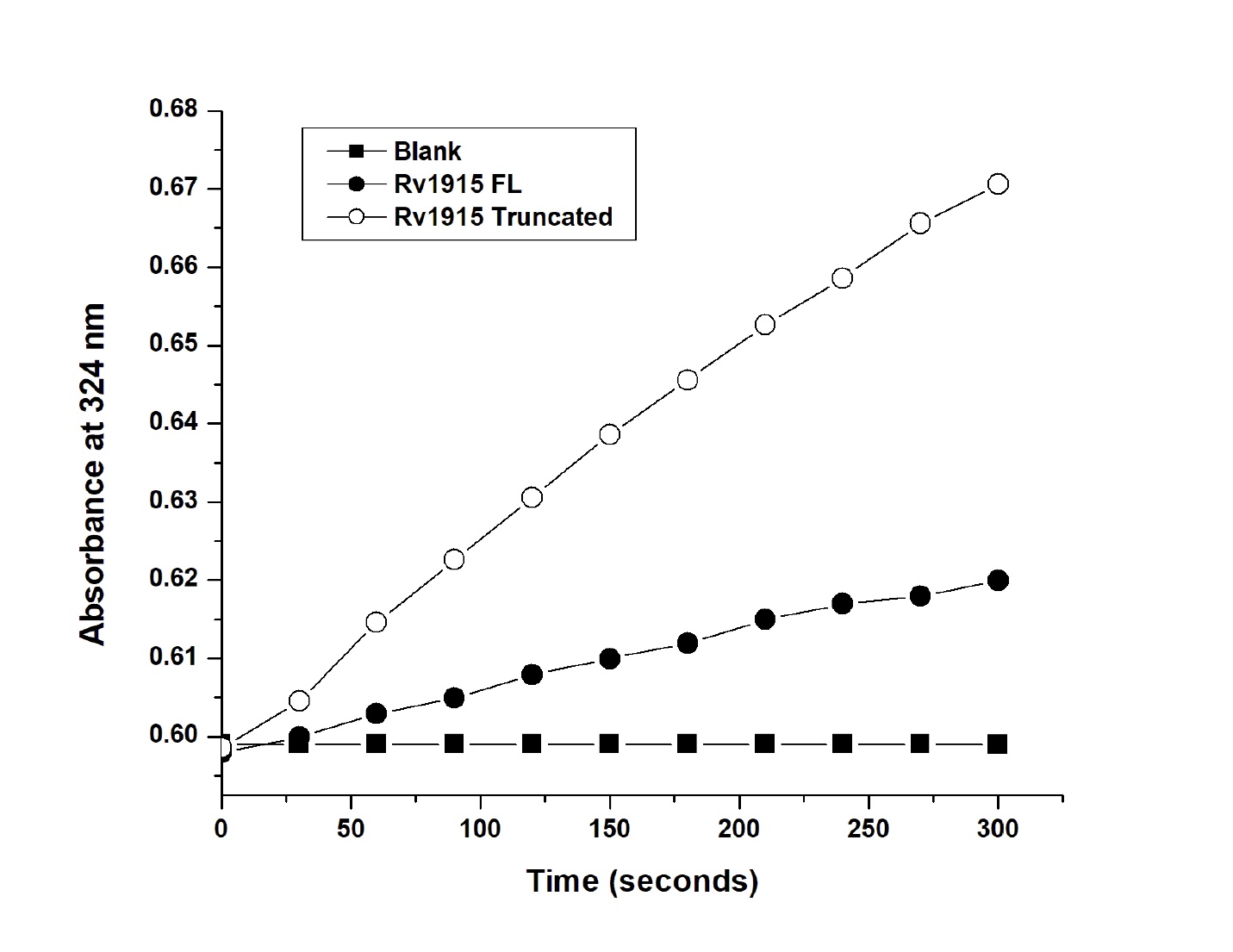
**

**Supplementary Figure 8: ICL activity of crude lysates of *E. coli* BL21 (DE3) over expressing His_6_-Rv1915 in comparison with Rv1915Δ90CT:** Time course curves represent the increase in absorbance due to the formation of isocitrate-glyoxylate phenylhydrazone complex monitored at 324nm.
